# Case Report: Hepatic Adenomatosis in a Patient With Prader–Willi Syndrome

**DOI:** 10.3389/fendo.2022.826772

**Published:** 2022-03-09

**Authors:** Hajar Dauleh, Ali Soliman, Basma Haris, Amal Khalifa, Noor Al Khori, Khalid Hussain

**Affiliations:** ^1^ Department of Pediatric Medicine, Division of Endocrinology, Sidra Medicine, Doha, Qatar; ^2^ Department of Pediatric Medicine, Division of Diagnostic Radiology, Sidra Medicine, Doha, Qatar

**Keywords:** hepatic adenomatosis, Prader Willi syndrom, liver adenoma, oral contraception pills, Glycogen Storage Disease

## Abstract

Prader–Willi syndrome (PWS) is a genetic disorder caused by the lack of expression of genes on the paternally inherited chromosome region 15q11.2-q13. It is a multisystem disorder that is characterized by severe hypotonia with poor suck and feeding difficulties in early infancy, followed in early childhood by excessive eating and gradual development of morbid obesity. The incidence of type 2 diabetes mellitus is high, particularly in obese patients. Non-alcoholic fatty liver disease has also been reported in some patients with PWS. Liver adenomatosis is a benign vascular lesion of the liver, defined by the presence of >10 adenomas, in the otherwise healthy liver parenchyma. We report the first case of a patient with PWS with severe obesity, type 2 diabetes mellitus, and non-alcoholic fatty liver who also developed liver adenomatosis, review the pediatric literature on liver adenomatosis, and discuss the potential underlying mechanisms.

## Introduction

Prader–Willi syndrome (PWS) is a complex multisystem disorder caused by lack of expression of genes on the paternally inherited chromosome region 15q11.2-q13. In the neonatal period, there is severe hypotonia with poor suck and feeding difficulties followed in infancy or early childhood by excessive eating and gradual development of morbid obesity. The syndrome is considered the most common genetic cause of obesity, occurring in 1:10,000–1:30,000 live births (1). Obesity and its related complications are the most common causes of morbidity and mortality in PWS. The mechanisms underlying the obesity include alterations in hypothalamic pathways that regulate satiety thus resulting in hyperphagia, disruption in hormones regulating appetite and satiety, and reduced energy expenditure (1).

Severe obesity is a strong risk factor for the development of type 2 diabetes mellitus (T2DM) in patients with PWS. T2DM in PWS occurs mostly in adults, but it has also been reported in patients under the age of 18 years (1). The prevalence of metabolic syndrome in obese patients with PWS seems to be similar to other obese patients (2). Patients with PWS are thought to be at a lower risk of developing non-alcoholic fatty liver disease (NAFLD) because of a higher insulin sensitivity as well as insulinopenia (3, 4) with the staging of the NAFLD depending on body composition (3, 4).

Liver adenomatosis is a benign vascular lesion of the liver, defined by the presence of >10 adenomas, in an otherwise healthy liver parenchyma (5). A variety of associations have been reported with liver adenomatosis including glycogen storage disease types I and IV, transfusion-induced hemosiderosis, Fanconi’s anemia, Hurler’s disease, severe combined immunodeficiency, familial adenomatous polyposis, and galactosemia (6).

We report the finding of liver adenomatosis in a case of PWS who is known to have severe obesity, T2DM, and NAFLD. We review all the previous reported cases of liver adenomatosis in pediatric patients and discuss the possible underlying mechanisms. Despite several cases reported in the literature, liver adenomatosis remains a poorly understood disease of unknown etiology.

## Case Presentation

We report a 17-year-old obese female patient with PWS who was born in Egypt and then moved to Qatar at the age of 9 years. She was born at term to non-consanguineous parents with a birth weight of 3.5 kg. The pregnancy was uneventful. In the newborn period, there were feeding difficulties, and on day 12 of life, she had a seizure with frothing, cyanosis, and jaw clenching. She was admitted for 10 days to the neonatal intensive care unit (NICU). There was a delay in achievement of motor milestones, with crawling starting at the age of 1 year 8 months and walking at 2 years. There was also a history of speech delay, and the patient started speaking at the age of 5 years. She started to gain weight from the age of 2 years; however, at the age of 12 years, around puberty, hyperphagia and excessive weight gain were noticed. Her parents had difficulty limiting her food intake, as she had a persistent feeling of hunger to the extent of vomiting after eating large quantities. She initially sought endocrine care at the age of 15 years for an obesity workup.

**Figure 1:**
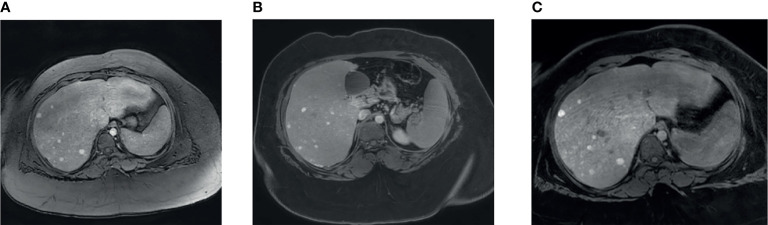
MRI liver findings. **(A)** Contrast enhanced liver MRI image showing multiple small liver lesions showing early arterial enhancement. **(B)** Lesions showing retention of contrast after 6 minute delay. **(C)** Lesions showing retention of contrast after 31 minute delay.

Ultrasound of the liver was done to rule out fatty liver, and this showed multiple lesions in the liver that were thought to be areas of focal fatty sparing. MRI of the liver was done that showed an enlarged liver with diffuse fat infiltration and multiple numerous focal lesions suggestive of multiple adenomas (adenomatosis; **Figure 1**). A follow-up ultrasound in 2021 showed that the liver remained markedly echogenic and heterogeneous. The largest measured adenoma was 13 mm superficially in the left lobe (previously up to 11 mm) and generally was of the order of around 6 mm in diameter. Echocardiography showed normal cardiac function and anatomy. The patient undergoes an MRI of the abdomen every 6 months for the liver Adenomatosis. To date, all MRI scans do not show any significant interval changes in the number or size of the adenomas.

During the coronavirus disease 2019 (COVID-19) quarantine, at the age of 16, she gained weight rapidly, with 17 kg in 1 year. The weight upon presentation was 124.1 kg (99.54 centile), height was 138.8 cm (0.01 centile: -3.73 Z-Score), and body mass index (BMI) was 64.4 kg/m^2^. The patient also had symptoms suggestive of sleep apnea with mouth breathing, snoring nights, and occasional daytime sleepiness. A sleep study revealed obstructive sleep apnea; hence, she was started on non-invasive ventilation. She had menarche at the age of 13, with irregular menses since then with prolonged heavy menses. In light of the long-term risks of endometrial hyperplasia and carcinoma due to her secondary amenorrhea, the oncology team recommended a minimum of four withdrawal bleeds per year to reduce the risk. The patient was prescribed medroxyprogesterone; she received a short course, 5 days, once every 3 months. She has received three courses since 2019 (15 days total in 2 years).

When she was 17 years of age, blood glucose values were high (250–300 mg/dl) during multiple clinic follow-ups with polyuria, polydipsia, and nocturia. Her blood gas did not show acidosis. HbA1c was 8.1% with high serum insulin and C-peptide levels along with acanthosis nigricans on the neck, axilla, and inguinal region suggestive of insulin resistance. The patient was started on both long-acting and short-acting insulin for glycemic control. **Table 1** shows the laboratory test results.

**Table 1:** Results of laboratory tests done.

Blood test	Blood test result	Reference range
Insulin level	618 pmol/L	20–571
C-peptide	14.9 ng/ml	(0.9–9.4)
HbA1c	8.1%	<6%
ALT	118 IU/L	9–22
AST	95 IU/L	15–28
ALP	141	48–96

ALT, Alanine Aminotransferase; AST, Aspartate Aminotransferase; ALP, Alkaline Phosphatase; HBA1c, Haemoglobin A1c.

In terms of management, metformin was started initially, but a response was not observed. Liraglutide, a glucagon-like peptide 1 receptor (GLP-1) agonist, was tried for a month to help with weight reduction and reduce insulin resistance. However, she developed a local skin allergy in the form of itchiness on the injection sites, so it was stopped. Her insulin regimen included long-acting insulin (Glargine) 20 units daily and rapid-acting insulin (noveorapid) of 3–4 units three times daily. Despite this, her obesity and T2DM were difficult to manage. A sleeve gastrectomy was done in October 2021, considering the extreme obesity, associated comorbidities, and the relatively poor response to medical therapy for her weight. Her 2-month post-sleeve gastrectomy weight is 113.2 kg (compared to 121 kg). Her blood glucose readings are within range, and she is off all antihyperglycemic medications.

### Family History

She is the youngest of three siblings; attended school for children with special needs. Father has T2DM.

### Genetic Testing

Methylation studies in 2019, when she was 15 years old, revealed an absence of the paternal allele at 15q11-q13 due to abnormal methylation, thus suggesting a defect in imprinting as the underlying molecular mechanism of the PWS.

### Radiology

MRI of the liver was done that showed an enlarged liver with diffuse fat infiltration and multiple numerous focal lesions suggestive of multiple adenomas (adenomatosis; **Figure 1**). A follow-up ultrasound in 2021 showed that the liver remained markedly echogenic and heterogeneous. The largest measured adenoma was 13 mm superficially in the left lobe (previously up to 11 mm) and generally was of the order of around 6 mm in diameter. Echocardiography showed normal cardiac function and anatomy.

## Discussion

Hepatic adenomas are benign vascular lesions of the liver that are usually solitary, associated with young women taking oral contraceptives. However, some patients have multiple adenoma, which is called liver adenomatosis. This was first described as a distinct entity in 1985 by Flejou et al. (7), who defined it as the presence of multiple adenomas (>10) that are not associated with steroid medication or glycogen storage disease. Currently, liver adenomatosis is defined as the presence of more than 10 adenomas in an otherwise normal parenchyma (8). The first clinical presentation due to liver adenomatosis is usually abdominal pain (9). Intraperitoneal bleeding, intratumoral hemorrhage, or necrosis producing acute pain are also reported (10). Only one report refers to malignant transformation of adenomas (11). This shows that even if the disease is benign, the risk of hemorrhage remains a concern.

We reviewed all the previous cases of liver adenomatosis in patients under the age of 18 years. **Table 2** summarizes the age of presentation, gender, clinical features of the patients, the use of oral contraceptives, and associated complications. No previous patients with PWS have been reported with liver adenomatosis. The mechanism behind the development of multiple hepatic adenomas is not well established, especially in the pediatric age group. Liver adenomatosis has been reported in association with metabolic conditions, vascular anomalies, with the use of oral contraceptives, and with inactivating mutations in the *HNF1A* gene.

**Table 2:** All pediatrics cases of LA reported in literature from years 1981 to 2019.

	Year/Author	Age in years	Associated conditions	Gender	OCP use	Clinical presentation	Complications
1	Flejou, 1981 (7)	13	None reported	M	No	Abdominal pain after trauma	Intraperitoneal bleeding from ruptured nodule
2	Chen, 1983 (12)	13	None reported	F	No	Hepatomegaly	None reported
3	Lesse, 1988 (11)	16	None reported	M	No	Abdominal pain due to ruptured nodule in the center lobe of the liver after trauma, found multiple adenomas	Malignant transformation
4	Kawakatsu, 1994 (13)	13	None reported	M	No	Pain, jaundice	Intratumoral
							bleeding
5	Gokhale, 1996 (14)	17	Minimal change GN diagnosed at the same time	F	No	Monthly abdominal pain and headache, resolve spontaneously	Hydronephrosis of the center kidney, cystic center ovary
6	Chiche, 2000 (8)	18	DM, non-insulin-dependent, hypertension on beta blockers	F	1year	Intraparietal bleeding	Extratumoral
							bleeding, cardiac arrest, and death
7	Chiche, 2000 (8)	14	None reported	M	No	Incidental finding	None reported
8	Chiche, 2000 (8)	17	None reported	F	No	Pain, hepatomegaly	Had segmentectomy, but 14 years later presented with intratumoral hemorrhage
9	Kadir Babaoglu 2010 (15)	7	CHD, Fontan procedure at 2 years of age	F	No	Abdominal distention	None reported
10	Wellen, 2010 (16)	15	None reported	F	Yes	Abdominal pain and weight loss	None reported
11	Timothy, 2019 (24)	15	Kapuki syndrome, medullary nephrocalcinosis, intermittent microhematuria, IgG deficiency, asthma, bilateral conductive hearing loss, and developmental delay	F	1 year	Intermittent abdominal pain, occasional dark urine in the preceding 2 months and unexplained pruritus for 10 years	HCC
12	Marino, 1992 (17)	10	Familial HA (mother and 8-year old brother were diagnosed with HA	F	No	Chronic recurrent episodes of abdominal pain	
13	Oji, 2019 (18)	18	Obesity, BMI 40	M	No	Incidental finding	None reported

OCP, oral contraceptive pills; HCC, hepatocellular carcinoma; GN, glomerulonephritis; HA, hepatic adenoma; CHD, congenital heart disease; ALT, Alanine Aminotransferase; AST, Aspartate Aminotransferase; ALP, Alkaline Phosphatase; HBA1c, Haemoglobin A1c.

With regard to metabolic disease, there is a strong association reported between liver adenomatosis and glycogen storage disease with 50%–80% of children with type I or III glycogen storage disease developing multiple hepatic adenomas (19, 20). The impairment in glycogenesis and the accumulation of glycogen deposits within the hepatocyte lead to hepatocyte hyperplasia, resulting in multiple adenoma formation. NAFLD is also reported to be associated with liver adenomatosis but mostly in adults (20). The increase in the intracellular lipid content could lead to a hyperplastic reaction with changes in oxidative and inflammatory pathways (21). An alternative mechanism of liver adenomatosis due to NAFLD suggests that the fatty tissue may generate continuous local estrogen through the increased activity of the enzyme aromatase, thus leading to the accelerated rate of hepatocyte growth and possible tumor formation (22). Multiple adenomas have been reported in other metabolic diseases such as diabetes, metabolic syndrome, and obesity in the adult population (8, 23).

The vascular hypothesis of liver adenomatosis is based on the association between reported cases of liver adenomatosis and hepatic vascular abnormalities, assuming that irregular vascular flow can result in the development of liver adenomatosis (**Table 2**). For instance, liver adenomatosis was reported in a 13-year-old male patient by Kawakatsu et al. (13) who had a spontaneous intrahepatic porto-hepatic venous shunt.

The association of liver cell adenomatosis and oral contraceptive or androgenic steroid use is still a point of controversy. Chiche et al. (8) reported that oral contraceptive therapy is not as rarely associated with this liver disease as initially suggested by Flejou et al. (7); 46% of their female patients were on oral contraceptives (9, 15). Adenoma regression was recognized after discontinuing hormonal contraceptives in multiple studies, which suggest that oral contraceptives play a role in the evolution of liver adenomatosis. In our review of the pediatric cases, we did not find a correlation with the use of oral contraceptives and liver adenomatosis, as only 3 out of 13 reported cases used oral contraceptives (**Table 2**).

In some patients with liver adenomatosis, there is a genetic background to the etiology. This involves biallelic inactivating mutations in the transcription factor, *HNF1A*, in the hepatic adenomas by the occurrence of two molecular events: either a germline and a somatic *HNF1A* mutation or two independent somatic events (25). This is usually associated with the Maturity Onset Diabetes of the Young (MODY) in the family history as heterozygous *HNF1A* mutations are a cause of MODY3.

Our patient with PWS has several risk factors for the development of liver adenomatosis. These include severe obesity (BMI of 64.4 mg/m^2^), T2DM, NAFLD, and possibly the use of oral contraceptives. Medroxyprogesterone was used for a short period of time, so we do not think that this is a significant risk factor, although we cannot completely rule this out. There was no family history of MODY in this patient. It is difficult to know which of these risk factors is directly linked to the liver adenomatosis but it is likely that the combination of the risk factors is involved. This case highlights that patients with PWS, who have risk factors such as obesity, T2DM, and NAFLD, may develop liver adenomatosis. Physicians should have a low threshold for making this possible diagnosis.

## Data Availability Statement

The original contributions presented in the study are included in the article/supplementary material. Further inquiries can be directed to the corresponding author.

## Author Contributions

HD and AS collected patient information, recruited the patient, analyzed and interpreted the data, and drafted the article. KH designed the study, obtained funding, and reviewed and edited the article. BH analyzed the data and reviewed and edited the article. All authors contributed to the article and approved the submitted version.

## Funding

This research was supported by the Qatar National Research Fund (QNRF-NPRP 10-6100017-AXX) awarded to KH.

## Conflict of Interest

The authors declare that the research was conducted in the absence of any commercial or financial relationships that could be construed as a potential conflict of interest.

## Publisher’s Note

All claims expressed in this article are solely those of the authors and do not necessarily represent those of their affiliated organizations, or those of the publisher, the editors and the reviewers. Any product that may be evaluated in this article, or claim that may be made by its manufacturer, is not guaranteed or endorsed by the publisher.

## References

[1] Muscogiuri G Barrea L Faggiano F Maiorino MI Parrillo M Pugliese G. Obesity in Prader-Willi Syndrome: Physiopathological Mechanisms, Nutritional and Pharmacological Approaches. J Endocrinol Invest (2021) 44(10):2057–70. doi: 10.1007/s40618-021-01574-9 PMC8421305 33891302

[2] Brambilla P Crinò A Bedogni G Bosio L Cappa M Corrias A. Metabolic Syndrome in Children With Prader-Willi Syndrome: The Effect of Obesity. Nutr Metab Cardiovasc Dis (2011) 21(4):269–76. doi: 10.1016/j.numecd.2009.10.004 20089384

[3] Talebizadeh Z Butler MG. Insulin Resistance and Obesity-Related Factors in Prader-Willi Syndrome: Comparison With Obese Subjects. Clin Genet (2005) 67(3):230–9. doi: 10.1111/j.1399-0004.2004.00392.x PMC6704480 15691361

[4] Haqq AM Muehlbauer MJ Newgard CB Grambow S Freemark M. The Metabolic Phenotype of Prader-Willi Syndrome (PWS) in Childhood: Heightened Insulin Sensitivity Relative to Body Mass Index. J Clin Endocrinol Metab (2011) 96(1):E225–32. doi: 10.1210/jc.2010-1733 PMC3038476 20962018

[5] Barbier L Nault JC Dujardin F Scotto B Besson M de Muret A. Natural History of Liver Adenomatosis: A Long-Term Observational Study. J Hepatol (2019) 71(6):1184–92. doi: 10.1016/j.jhep.2019.08.004 31419515

[6] Davenport M Pakarinen M. Preface - Pediatric Hepatobiliary Surgery. Semin Pediatr Surg (2020) 29(4):150944. doi: 10.1016/j.sempedsurg.2020.150944 32861454

[7] Flejou JF Barge J Menu Y Degott C Bismuth H Potet F. Liver Adenomatosis. An Entity Distinct From Liver Adenoma? Gastroenterology (1985) 89(5):1132–8. doi: 10.1016/0016-5085(85)90220-3 2412930

[8] Chiche L Dao T Salamé E Galais MP Bouvard N Schmutz G. Liver Adenomatosis: Reappraisal, Diagnosis, and Surgical Management: Eight New Cases and Review of the Literature. Ann Surg (2000) 231(1):74–81. doi: 10.1097/00000658-200001000-00011 10636105 PMC1420968

[9] Ribeiro A Burgart LJ Nagorney DM Gores GJ. Management of Liver Adenomatosis: Results With a Conservative Surgical Approach. Liver Transpl Surg (1998) 4(5):388–98. doi: 10.1002/lt.500040505 9724476

[10] Lui AF Hiratzka LF Hirose FM. Multiple Adenomas of the Liver. Cancer (1980) 45(5):1001–4. doi: 10.1002/1097-0142(19800301)45:5<1001::aid-cncr2820450528>3.0.co;2-f 7260831

[11] Leese T Farges O Bismuth H. Liver Cell Adenomas. A 12-Year Surgical Experience From a Specialist Hepato-Biliary Unit. Ann Surg (1988) 208(5):558–64. doi: 10.1097/00000658-198811000-00003 PMC1493795 3190282

[12] Chen KT Bocian JJ. Multiple Hepatic Adenomas. Archives of Pathology & Laboratory Medicine (1983) 107(5):274–5. 6301400

[13] Kawakatsu M Vilgrain V Belghiti J Flejou JF Nahum H. Association of Multiple Liver Cell Adenomas With Spontaneous Intrahepatic Portohepatic Shunt. Abdom Imaging (1994) 19(5):438–40. doi: 10.1007/BF00206934 7950822

[14] Gokhale R Whitington PF. Hepatic Adenomatosis In an Adolescent. J Pediatr Gastroenterol Nutr (1996) 23:482–6. 10.1097/00005176-199611000-00024 8956193

[15] Babaoglu K Binnetoglu FK Aydoğan A Altun G Gürbüz Y Inan N. Hepatic Adenomatosis in a 7-Year-Old Child Treated Earlier With a Fontan Procedure. Pediatr Cardiol (2010) 31(6):861–4. doi: 10.1007/s00246-010-9685-x 20204345

[16] Wellen JR Anderson CD Doyle M Shenoy S Nadler M Turmelle Y. The Role of Liver Transplantation for Hepatic Adenomatosis in the Pediatric Population: Case Report and Review of the Literature. Pediatr Transplant (2010) 14(3):E16–9. doi: 10.1111/j.1399-3046.2008.01123.x 19490491

[17] Marino IR Scantlebury VP Bronsther O Iwatsuki S Starzl TE. Total Hepatectomy and Liver Transplant for Hepatocellular Adenomatosis and Focal Nodular Hyperplasia. Transpl Int (1992) 5(Suppl 1):S201–5. doi: 10.1007/978-3-642-77423-2_64 PMC2993572 14621777

[18] Oji K Urade T Iwatani Y. Case of Resected Multiple Hepatocellular Adenomas in a Young Man with Severe Obesity. Surg Case Rep (2019) 5:131. 31410698 10.1186/s40792-019-0689-3 PMC6692803

[19] Barthelmes L Tait IS. Liver Cell Adenoma and Liver Cell Adenomatosis. HPB (Oxford) (2005) 7(3):186–96. doi: 10.1080/13651820510028954 PMC2023950 18333188

[20] Brunt EM Wolverson MK Di Bisceglie AM. Benign Hepatocellular Tumors (Adenomatosis) in Nonalcoholic Steatohepatitis: A Case Report. Semin Liver Dis (2005) 25(2):230–6. doi: 10.1055/s-2005-871202 15918151

[21] Powell EE Jonsson JR Clouston AD. Steatosis: Co-Factor in Other Liver Diseases. Hepatology (2005) 42(1):5–13. doi: 10.1002/hep.20750 15962320

[22] Wake DJ Strand M Rask E Westerbacka J Livingstone DE Soderberg S. Intra-Adipose Sex Steroid Metabolism and Body Fat Distribution in Idiopathic Human Obesity. Clin Endocrinol (Oxf) (2007) 66(3):440–6. doi: 10.1111/j.1365-2265.2007.02755.x 17302881

[23] Furlan A van der Windt DJ Nalesnik MA Sholosh B Ngan KK Pealer KM. Multiple Hepatic Adenomas Associated With Liver Steatosis at CT and MRI: A Case-Control Study. AJR Am J Roentgenol (2008) 191(5):1430–5. doi: 10.2214/AJR.07.3419 18941081

[24] Timothy LD Lehrke HD Chandan VS Kolbe AB Furuya KN. Diffuse Adenomatosis and Hepatocellular Carcinoma Treated With Liver Transplantation in an Adolescent Female With Kabuki Syndrome With a Novel KMT2D Gene Mutation. Case Rep Pediatr (2019) 2019:7983824. doi: 10.1155/2019/7983824 31179148 PMC6507262

[25] Bluteau O Jeannot E Bioulac-Sage P Marqués JM Blanc JF Bui H. Bi-Allelic Inactivation of TCF1 in Hepatic Adenomas. Nat Genet (2002) 32(2):312–5. doi: 10.1038/ng1001 12355088

